# Similarity Effect and Purchase Behavior of Organic Food Under the Mediating Role of Perceived Values in the Context of COVID-19

**DOI:** 10.3389/fpsyg.2021.628342

**Published:** 2021-10-13

**Authors:** Chunnian Liu, Yan Zheng, Dayu Cao

**Affiliations:** ^1^School of Management, Nanchang University, Nanchang, China; ^2^School of Economics and Management, Jiangxi Agricultural University, Nanchang, China; ^3^School of Economics and Management/Jiangxi Rural Revitalization Strategy Research Institute, Jiangxi Agricultural University, Nanchang, China

**Keywords:** SOR model, organic food, perceived values, sustainable consumption, information similarity effect

## Abstract

Due to the influence of COVID-19, people pay more attention to the balance between human and nature and pursue more healthy, environmental and nutritional sustainable products (such as organic food). However, the mainstream consumption of organic food is far less, especially in developing countries like China. Therefore, it is urgent to take effective measures to promote the development of China's organic food market. This current study investigated the relationships between consumers' similarity (i.e., information anxiety, uncertainty, and sustainable consumption attitude), perceived values (i.e., functional value, health value, and environmental value) and organic purchasing behavior based on the Stimulus-Organism-Response (S-O-R) theoretical model and information similarity effect. And considering gender differences in consumers' similarity, perceived values and organic purchasing behavior. Meanwhile, the mediating effects of perceived values on the relationship between consumers' similarity and purchasing behavior were also discussed, considering the background of COVID-19. Data were collected using structured questionnaire survey in first-tier cities in China. A total of 344 consumers of organic foods participated in the study. Structural equation modeling was employed for data analysis. The results indicated the significant association of information anxiety, uncertainty and sustainable consumption attitude with perceived values. And perceived values and sustainable consumption attitude had a positively significant influence on purchase behavior. In addition, environmental value played mediating effects in the relationships between organic purchasing behavior and information anxiety, uncertainty and sustainable consumption attitude. And the impact of sustainable consumption attitude and environmental value on organic purchasing behavior differed in gender. The research not only provides novel insights for understanding organic consumption, but also provides reference for organic sellers to develop sales strategies and policy makers to formulate policies to guide organic consumption, which are conducive to promoting China's organic food industry.

## Introduction

Global food safety and environmental problems have attracted more and more attention. Thøgersen (2017) believed that promoting sustainable food consumption would be the key to alleviate and improve a series of environmental and health problems. As organic food is healthier and more environmentally friendly than traditional food and can support the local economy (Strassner et al., 2015; Verain et al., 2015; De-Magistris and Gracia, 2016), the consumption of organic food is considered as an important form of sustainable consumption (Strassner et al., 2015; Seconda et al., 2017). The organic market has grown rapidly as the public pays more attention to healthy, safe, nutritious and environmentally friendly organic foods (Kareklas et al., 2014; Basha and Lal, 2019). In 2018, the total global market value of organic food was estimated to be 96.7 billion euros, and global per capita consumption was approximately 12.8 euros (Willer and Lernoud, 2020). By 2018, China had already become the world's third-largest organic food market (8.1 billion euros, 8.3% of the global market) (Willer and Lernoud, 2020). However, China's per capita consumption is only approximately 5.8 euros, less than half the level of global per capita consumption. In particular, its organic food consumption is far from that of top-ranked Switzerland (per capita consumption of approximately 312 euros) (Willer and Lernoud, 2020). In addition, China's organic food market started late, the organic food market is still relatively small in size at present, and consumer groups are mainly concentrated in large cities (Xu, 2017), which hinders the development of China's organic industry.

Understanding organic purchasing behavior is the key to predicting organic food consumption (Rana and Paul, 2017). Many scholars have done a lot of research on the influencing factors of organic consumption behavior. Some studies believed that consumer perceived values play important roles in promoting the purchase of organic food (Suki and Suki, 2015a,b,c; Suki, 2016; Akbar et al., 2019; Kushwah et al., 2019a; Shamsi et al., 2020). In particular, health value, functional value and environmental value were identified as important factors in predicting organic purchasing behavior (Mohammed, 2020). Chekima et al. (2017) found that consumers' attitudes toward organic food can play a positive role in organic consumption. At the same time, Lockie et al. (2004) and Stobbelaar et al. (2007) showed that there are gender differences in organic purchasing. Stevens (2020) and Newburn (2020) pointed out that, especially in the context of COVID-19, the impact of consumer's gender differences on behavior and attitude is more and more obvious. In addition, asymmetric information in organic market, uncertainty and insufficient marketing were identified as the key factors hindering consumers to buy organic food (Teng and Lu, 2016; Hidalgo-Baz et al., 2017; Liu and Zheng, 2019; Kongtip et al., 2020; Wang et al., 2020; Xie et al., 2020). With the development of new media, consumers get more and more information from various media. Some studies believed that the more organic information consumers get from the media, the more they tend to buy organic food, which can improve the information asymmetry in the organic market (De-Magistris and Gracia, 2016; Liu et al., 2021).

However, due to the great differences of information about organic food obtained by consumers from different information sources, the increase of information sources may hinder consumers' organic purchase. Fu et al. (2018) believed that consumers' information similarity can affect their perception and purchase behavior. Similarity generally includes genetic, social, cultural, physical, and psychological factors (Thøgersen, 2004; Hitsch et al., 2010). In addition, Guéguen et al. (2011) proved that similarity can affect people's internal perception. Therefore, it is not the number of information sources, but the information similarity of different information sources that is important to consumers' purchasing behavior.

With the outbreak of COVID-19 in China and the rest of the world, the epidemic has spread quickly, and the situation is serious (Jia et al., 2020). Consumers can get the similar information through various media every day, so how does the information similarity affect the organic purchase behavior? Therefore, based on the Stimulus-Organism-Response (S-O-R) theoretical model and information similarity effect, the current study explored the relationships between consumers' similarity, perceived values and organic purchasing behavior. And considering gender differences in consumers' similarity, perceived values and organic purchasing behavior. Meanwhile, the mediating effects of perceived values on the relationship between consumers' similarity and purchasing behavior were also discussed, considering the background of COVID-19.

This paper intends to make three contributions to the literature. First, previous studies rarely focused on the relationship between consumer information similarity and organic purchasing behavior. In order to fill this gap, this paper explored the relationship between consumer information similarity and the organic purchasing behavior in the context of COVID-19, which may enrich the research of organic consumption. Second, based on the SOR model and information similarity effect, this paper divided stimulus (S) factors into external similarity and internal similarity. In addition, this paper divided external similarity into information anxiety and uncertainty from the perspective of consumers' access to information, which not only considered the similarity of information anxiety, but also considered consumers' similarity to the uncertainty of organic information. This may provide a new perspective for the study of organic consumption, and may provide valuable suggestions for organic sellers. Third, this paper focused on the role of consumer perceived values after the COVID-19 outbreak, divided perceived values into three dimensions of functional value, health value, and environmental value in combination with the realistic background, and explored the relationship between perceived values and organic purchasing behavior. At the same time, the mediating effects of perceived values on the relationship between information anxiety, uncertainty, sustainable consumption attitude, and purchasing behavior were also discussed. The conclusions may provide valuable suggestions for organic retailers, policy makers and even organic producers.

The rest of the paper is organized as follows. In Sections Theoretical Background and Hypothesis Development, we review the background literature and develop our hypotheses. Sections Research Methodology and Results provide a detailed introduction of our research methodology and analysis and present our research results. Finally, we discuss some conclusions, significance and limitations of this study, as well as ideas for further research.

## Theoretical Background

### The Stimuli-Organism-Response Model (SOR)

According to the SOR model from the field of environmental psychology, all aspects of the environment play a stimulating role (S), affecting people's internal states (O), which drives their behavioral responses (R) (Mehrabian and Russell, 1974). The model shows that external environmental factors affect the psychological changes of organisms, thus prompting them to adopt behavioral responses. Meanwhile, it also explains the change of people's internal state strengthened by the stimulation of external elements (Eroglu et al., 2001). Previous studies have shown that people's inner state has both positive and negative effects (Verhagen and van Dolen, 2011). Finally, People make the final choice according to the internal state and take corresponding behavioral responses (Mehrabian and Russell, 1974).

The SOR model is applicable to the present study for the following two reasons. Firstly, the SOR model has been extensively used in prior studies consumers' behaviors (Parboteeah et al., 2009; Wang et al., 2011; Luqman et al., 2017; Fu et al., 2018; Li and Yuan, 2018). For example, Luqman et al. (2017) applied the SOR model to social media to identify the user's behavior and its consequences. Fu et al. (2018) studied whether environmental stimulation can improve users' willingness to buy movie tickets online based on SOR model. Secondly, in view of the important role of environmental factors in influencing consumer behavior, the SOR model provides a concise and structured way to test the impact of environmental stimulus on consumer psychological factors (e.g., emotion, perception, and cognition), and then test the impact of consumers on organic purchasing behavior. Therefore, the current study applies this model to consumer behavior.

#### Stimuli (S)

Stimulus refers to all kinds of environmental factors encountered by individuals (Jacoby, 2002). Previous studies on consumer behavior showed that consumers obtain information through various news media and interact with others through social media (Hajli, 2014). Over time, consumers may discover factors that they are similar to each other (e.g., interest, attitude, and preference), thus stimulating their internal perception and ultimately generating behavioral response (Fu et al., 2018). Therefore, the similarity of consumers is an important stimulus factor affecting consumers' internal perception and behavior.

#### Organism (O)

Organism refers to the internal perception of consumers (Eroglu et al., 2001). Perceived values are important parts of internal perception. They are the internal driving force for consumers to choose a certain product and an important indicator to predict consumers' purchasing behavior (Fu et al., 2018). Sheth et al. (1991) put forward the theoretical framework of perceived values. They believed that perceived values include five dimensions: functional value, social value, emotional value, epistemic value, and conditional value. Rahnama (2016) predicted consumers' organic purchasing behavior through seven dimensions of perceived values: functional value, health value, environmental value, epistemic value, social value, emotional value and conditional value. Referring to the above research, according to the theme of the current study, we constructed three dimensions of perceived values: functional value, health value and environmental value to explore organic consumption behavior.

#### Response (R)

Response is the final result and decision of consumers based on internal perception, including approach or avoidance behavior (Sherman et al., 1997). In essence, consumers' organic purchase behavior can be regarded as the approach behavior made by consumers based on internal perception. Therefore, the present paper considered the impact of consumer perceived values on consumers' organic purchasing behavior.

### The Similarity Effect

In sociology, we describe the similarity effect as people's strong preference for people with similar characteristics. The concept of similarity has been widely studied in psychology and consumer behavior. Based on the hypothesis that similarity causes changes in internal perception (Byrne, 1971), many scholars have studied the similarity of different personal attributes, such as demographic information (Hitsch et al., 2010), wearing the same clothes (Buckley and Roach, 1981), and the same attitude (Thøgersen, 2004). Fu et al. (2018) believed that similarity includes genetic, social, cultural, physiological and psychological factors. Meanwhile, Guéguen et al. (2011) proved that similarity can affect people's internal perception.

Fu et al. (2018) divided the similarity into external similarity and internal similarity. External similarity includes demographic information and various media information, while internal similarity includes interest, attitude or opinion, preference, etc. In this study, we divided external similarity into two dimensions of information anxiety and uncertainty from the perspective of consumers' access to information. This is mainly because on the one hand, under the severe influence of COVID-19 in China (Jia et al., 2020), consumers can obtain a lot of information about COVID-19 through media every day. Therefore, we believed that consumers may generally have information anxiety factors. On the other hand, China's organic market is in the initial stage of development, and there is information asymmetry in the market (Xu, 2017). Therefore, we divided external similarity into two dimensions of information anxiety and uncertainty, which may not only provide a new perspective for organic consumption research, but also provide valuable suggestions for organic sellers.

In addition, this study took sustainable consumption attitude as an internal similarity. This is because existing studies have shown that COVID-19 is caused by ecological imbalance (Lvov and Alkhovsky, 2020), which urges people to pay more attention to ecological balance and pursue healthy and environmentally sustainable consumption.

## Hypothesis Development

**Figure 1:**
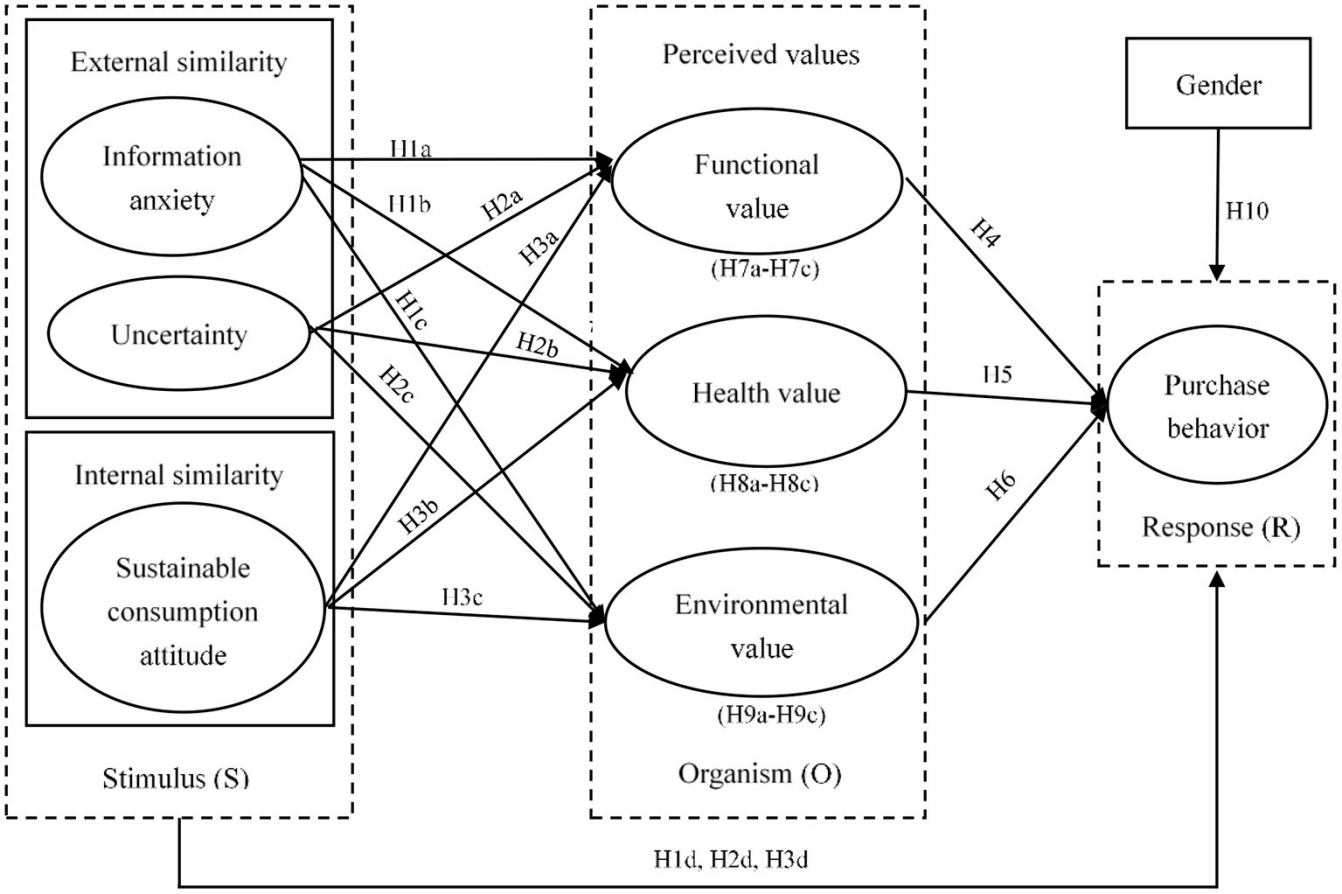
Research framework.

This study aims to examine the impact of similarity on consumers' organic purchasing behavior from the perspective of consumer perceived values based on SOR model. Figure 1 depicts the research framework, which reflects the influence of external similarity (i.e., information anxiety and uncertainty) and internal similarity (i.e., sustainable consumption attitude) on organic purchase behavior, as well as the role of perceived values (i.e., functional value, health value and environmental value). In this section, we explain the primary constructs and interrelationships in the research model.

### The Effect of Information Anxiety on Perceived Values and Purchase Behavior

With the outbreak of COVID-19 in China and the rest of the world, the epidemic has spread quickly, and the situation is serious (Jia et al., 2020; Xie et al., 2020). Consumers can get a lot of information about COVID-19 through media every day. As time goes on, consumers may have information anxiety in common. And Shweta et al. (2017) confirmed that people often contact with massive certain information, and over time, people would find that they have common information anxiety characteristics. Ruiz Mafé and Sanz Blas (2006) found that the information that consumers are exposed to through various information channels can affect their perceived values and behavior. Previous studies on perceived values have divided it into different dimensions according to different research topics. For example, Rahnama (2016) divided perceived values into seven dimensions: functional value, health value, environmental value, epistemic value, social value, emotional value and conditional value. Gonçalves et al. (2016) divided perceived values into five dimensions: functional value, social value, emotional value, conditional value and epistemic value. Referring to the above research, according to the theme of this paper, we construct three dimensions of perceived values: functional value, health value, and environmental value. Therefore, this study proposes the following hypothesis:

H1a. Information anxiety (IA) has a positive effect on functional value (FV).
H1b. Information anxiety (IA) has a positive effect on health value (HV).
H1c. Information anxiety (IA) has a positive effect on environmental value (EV).
H1d. Information anxiety (IA) has a positive effect on purchase behavior (PB).

### The Effect of Uncertainty on Perceived Values and Purchase Behavior

Uncertainty is a state of holding incomplete information about something (Vieira, 2008), and it is considered to have a negative impact on consumers' perceived values and purchase intention (Shiu et al., 2011). Previous studies have shown that the lack of relevant information and understanding of organic labels would increase the difficulty for consumers to distinguish the credence attributes and standards from that of traditional foods (Magistris and Gracia, 2008; Janssen and Hamm, 2011). Moreover, some studies argued that uncertainty toward the real attributes of organic food has a negative influence on consumers' perceived values and purchase intention (Yiridoe et al., 2005; Nuttavuthisit and Thogersen, 2017). Thus, we propose the following hypothesis:

H2a. Uncertainty (UNC) has a negative effect on functional value (FV).
H2b. Uncertainty (UNC) has a negative effect on health value (HV).
H2c. Uncertainty (UNC) has a negative effect on environmental value (EV).
H2d. Uncertainty (UNC) has a negative effect on purchase behavior (PB).

### The Effect of Sustainable Consumption Attitude on Perceived Values and Purchase Behavior

Attitude plays an important role in influencing consumers' perceived values and behavior (Follows and Jobber, 2000; Hidalgo-Baz et al., 2017). Understanding consumer attitudes can help policy makers, marketers and producers promote sustainable consumption habits and encourage consumers to consume or use green products (Lin and Huang, 2012). In addition, Marchand and Walker (2008) pointed out that consumers' attitude toward sustainable consumption can promote the change of their perceived values, so as to seek a more sustainable lifestyle. Therefore, we propose the following hypothesis:

H3a. Sustainable consumption attitude (SCA) has a positive effect on functional value.
H3b. Sustainable consumption attitude (SCA) has a positive effect on health value.
H3c. Sustainable consumption attitude (SCA) has a positive effect on environmental value.
H3d. Sustainable consumption attitude (SCA) has a positive effect on purchase behavior (PB).

### The Effect of Perceived Values on Purchase Behavior

Perceived values refer to consumers' overall evaluation of the usefulness of relevant products (Zeithaml, 1988). Perceived values are considered important predictors of consumer decision-making (Sheth et al., 1991). Moreover, perceived values can explain the internal reasons for consumers to choose specific products (Sheth et al., 1991). According to different research topics, scholars divided perceived values into different dimensions. For example, Rahnama (2016) divided perceived values into seven dimensions: functional value, health value, environmental value, epistemic value, social value, emotional value, and conditional value. Therefore, according to the research theme of this paper, we construct the perceived values of three dimensions: functional value, health value, and environmental value. As such, we propose the following hypothesis:

H4. Functional value has a positive effect on purchase behavior (PB).
H5. Health value has a positive effect on purchase behavior (PB).
H6. Environmental value has a positive effect on purchase behavior (PB).

### Mediating Effect of Perceived Values

Perceived values are considered important predictors of consumer decision-making (Sheth et al., 1991). If the attribute of a product is related to the individual's consumption motivation, the individual experience will be stimulated by a certain degree of cognitive or emotional arousal, which will trigger the individual's perception of the product and make it related to him or her (Frieze, 1997). Lin and Huang (2012) found that organic purchasing behavior is not only directly affected by consumption motivation, but also affected by one's perceived values of organic food. Previous studies have shown that perceived values and belief mediate the relationship between consumer motivation and organic buying (Çabuk et al., 2014; Pagiaslis and Krontalis, 2014; Wang et al., 2019). As COVID-19 has been for a period of time around the world, consumers' information anxiety about COVID-19 may indirectly affect organic purchasing behavior through perceived values. In addition, Lvov and Alkhovsky (2020) found that COVID-19 is caused by the destruction of ecological balance, so people's attitude toward sustainable consumption may also indirectly affect organic purchasing behavior through perceived values. Therefore, it can be postulated that perceived values are mediators linking the relationship between organic purchasing behavior and information anxiety, uncertainty and sustainable consumption attitude. Accordingly, the hypotheses are proposed as follows:

H7a-c. Functional value mediates the effect of information anxiety, uncertainty, and sustainable consumption attitude on purchase behavior respectively.
H8a-c. Heath value mediates the effect of information anxiety, uncertainty, and sustainable consumption attitude on purchase behavior respectively.
H9a-c. Environmental value mediates the effect of information anxiety, uncertainty, and sustainable consumption attitude on purchase behavior respectively.

### Gender and Purchase Behavior

Previous studies have shown that gender influences the organic purchasing behavior. Lockie et al. (2004) found that women hold a higher proportion of positive attitudes toward organic food than do men. Stobbelaar et al. (2007) found that adolescent girls show a higher preference for organic products than do boys. Stevens (2020) and Newburn (2020) pointed out that, especially in the context of COVID-19, the impact of consumer's gender differences on behavior and attitude is more and more obvious. Thus, we propose the following:

H10. The organic purchase behavior of consumers differs according to consumer gender.

## Research Methodology

### Data Sources

Data collection was conducted through the commission of a professional online questionnaire service company, Wenjuanxing (https://www.wjx.cn). And in the present study, respondents from Beijing, Shanghai, Guangzhou and Shenzhen were selected as the research objects. There are two main reasons for using online surveys to collect respondents from these cities in the current study. First, face-to-face interviews should be avoided to reduce social distance after the COVID-19 outbreak. Second, the price of organic food is usually 2–4 times higher than that of traditional agricultural products, and China's organic consumers are mainly concentrated in big cities at present (Xu, 2017). According to the China Statistical Yearbook 2020 (http://www.stats.gov.cn/tjsj/ndsj/), these cities were the top-four-ranked cities for per capita disposable income in China. Therefore, the samples from Beijing, Shanghai, Guangzhou, and Shenzhen are more representative. Moreover, 30 questionnaires were distributed online prior to the formal survey as a presurvey to ensure the comprehensibility of the items in the survey and the appropriateness of data collection procedures. After the preliminary survey, the questionnaire was modified appropriately.

In addition, we included the following question in the questionnaire: “Have you ever bought organic food before?” In answering this question, 16 respondents chose “no.” Because the current study was about consumers' organic purchasing behavior, 16 respondents were excluded. Thus, we ultimately obtained 344 usable responses out of the 360 initial responses. As shown in Table 1, there were 189 male (54.9%) and 155 female (45.1%) respondents in the collected sample. There were 293 respondents (85.2%) with a junior college or an undergraduate education in the sample. The respondents aged between 18 and 40 accounted for 41.9% of the total sample. Respondents with a per capita monthly income of more than ¥5,000 accounted for 89.5% of the sample.

**Table 1:** Demographic profile of the sample (*N* = 344).

	***n***	**%**
1. Gender		
Male	189	54.9
Female	155	45.1
2. Age		
18–30	144	41.9
31–40	151	43.9
41–50	42	12.2
>50	7	2
3. Education		
Junior high school and below	2	0.6
High school or technical secondary school	16	4.7
Junior college or undergraduate	293	85.2
Postgraduate and above	33	9.6
4. Per capita monthly income		
<¥3,000	2	0.6
¥3,001–¥5,000	34	9.9
¥5,001–¥8,000	79	23
¥8,001–¥12,000	115	33.4
>¥12,000	114	33.1
5. Distribution area of respondents		
Beijing	113	32.8
Shanghai	138	40.1
Guangdong Province (including only Guangzhou and Shenzhen)	93	27

### Measures

All constructs in the proposed model were measured with multiple-item scales that were validated in previous studies. A few minor modifications were made to the measures to ensure that they had face validity in the current research context. The items used a 5-point Likert scale ranging from “1 = strongly disagree” to “5 = strongly agree” (see Appendix A). Five items of information anxiety were adapted from Zung (1971) and Shweta et al. (2017); four items of uncertainty were adapted from Kushwah et al. (2019b); four items of sustainable consumption attitude were adapted from Arvola et al. (2008) and Dean et al. (2012); seven items of functional value were adapted Kushwah et al. (2019a) and Akbar et al. (2019); three items of health value were adapted from Rahnama (2016); four items of environmental value were adapted from Biswas and Roy (2015); and the three items of purchase behavior were adapted from Michaelidou and Hassan (2008) and Singh and Verma (2017).

### Analytical Method

To test the proposed model, we adopted the two-stage approach of “structural equation modelling” (SEM) recommended by Anderson and Gerbing (1988). AMOS 24.0 was used for assessing the model fit as well as for hypothesis testing. In addition, to assure construct validity, we also compute Confidence Interval for Cronbach's alpha according to Trinchera et al. (2018) suggestions using R 3.5.1.

### Common Method Bias

As with all self-reported data, there is a potential for common method variance resulting from multiple sources, such as consistency motif and social desirability (Podsakoff et al., 2003). Several techniques can be used to detect and control common method bias, such as measured marker variables (correlation-based, regression-based, and CFA-based) and unmeasured latent method factors (Podsakoff et al., 2003, 2012). We took Harman's single-factor test, as suggested by Podsakoff et al. (2003), to address concerns regarding common method bias. All measurement items were subjected to exploratory factor analysis using SPSS 23.0. The unrotated factor solutions revealed that the single factor explained only 29.26% of the variance in the variables. Therefore, we can conclude that common method bias is unlikely to be a serious concern for this study.

The seven constructs of IA, UNC, SCA, FV, HV, EV, and PB were measured by the concept of reflective indicators.

## Results

### Validity of Measurement Model

Confirmatory factor analysis (CFA) assesses the fit of the measurement model based on various fit indices. According to the guidelines suggested by Jackson et al. (2009), the chi-square (χ^2^) value, degrees of freedom (df), value of χ^2^/df, comparative fit index (CFI), root mean square error of approximation (RMSEA), goodness-of-fit index (GFI), and Tucker-Lewis index (TLI) were used to assess model fit. The model fit is good when χ^2^/df < 3.0, with RMSEA ≤ 0.08, TLI and CFI ≥ 0.90 (Hu and Bentler, 1999), and GFI ≥ 0.80 (Chau and Hu, 2001). However, a few model fit statistics were not greater than their minimum acceptable level in the study, which is due to the expansion of the chi-square value caused by nonmultivariate normality (Enders, 2005). Thus, the Bollen–Stine bootstrap was used to correct for bias in the model fit statistic (Bollen and Stine, 1992; Fisher and King, 2010). According to a Bollen–Stine bootstrap with 2000-times correction, the resultant fit statistics (χ^2^ = 451.73; df = 387; χ^2^/df = 1.17; CFI = 0.99; GFI = 0.93; TLI = 0.99; RMSEA = 0.02) were all acceptable.

To measure the internal consistency reliability, convergent validity and discriminant validity of the constructs in our proposed model, we performed CFA analysis on the eight constructs of IA, UNC, SCA, FV, HV, EV, and PB (see Tables 2, 3). The results revealed that the values for both Cronbach's alpha and composite reliability (CR) were over 0.7, and thus internal consistency reliability was acceptable (Nunnally, 1978). In addition, the factor loadings of the individual items in the eight-construct model were all significant (all *p* < 0.001), indicating preliminary evidence for the convergent validity of the measurement model (Diamantopoulos et al., 2008). Meanwhile, the average variance extracted (AVE) of all constructs exceeded the 0.5 AVE threshold value (Fornell and Larcker, 1981; Bagozzi and Yi, 1989), and thus the convergent validity was acceptable. Moreover, Table 4 shows that the estimated intercorrelations among all constructs were less than the square roots of the AVE in each construct, and this provides support for discriminant validity (Fornell and Larcker, 1981).

**Table 2:** Coefficients for the measurement model.

**Construct**	**Variable**	**Unstd. Estimates**	**S.E**.	***T*-value**	**Std. factor**	**CR**	**AVE**
					**Loadings**		
Information anxiety	IA1	1.000	—	—	0.773	0.901	0.646
	IA2	1.078	0.066	16.453^***^	0.841		
	IA3	0.994	0.062	16.114^***^	0.826		
	IA4	1.005	0.063	15.832^***^	0.814		
	IA5	0.907	0.062	14.633^***^	0.761		
Uncertainty	UNC1	1.000	—	—	0.693	0.819	0.531
	UNC2	1.093	0.095	11.517^***^	0.722		
	UNC3	1.187	0.096	12.373^***^	0.796		
	UNC4	0.977	0.087	11.214^***^	0.699		
Sustainable	SCA1	1.000	—	—	0.677	0.837	0.563
consumption attitude	SCA2	1.114	0.095	11.677^***^	0.735		
	SCA3	1.278	0.101	12.622^***^	0.815		
	SCA4	1.126	0.093	12.085^***^	0.767		
Functional value	FV1	1.000	—	—	0.613	0.859	0.501
	FV2	1.140	0.117	9.746^***^	0.639		
	FV3	1.118	0.115	9.717^***^	0.637		
	FV4	1.049	0.108	9.691^***^	0.634		
	FV5	1.289	0.125	10.354^***^	0.693		
	FV6	1.451	0.129	11.243^***^	0.780		
	FV7	1.564	0.140	11.186^***^	0.774		
Health value	HV1	1.000	—	—	0.808	0.851	0.656
	HV2	0.935	0.063	14.775^***^	0.772		
	HV3	1.040	0.065	16.062^***^	0.848		
Environmental value	EV1	1.000	—	—	0.868	0.894	0.680
	EV2	1.013	0.052	19.335^***^	0.837		
	EV3	0.828	0.054	15.215^***^	0.715		
	EV4	1.021	0.050	20.415^***^	0.868		
Purchase behavior	PB1	1.000	—	—	0.760	0.819	0.602
	PB2	1.292	0.097	13.336^***^	0.791		
	PB3	1.261	0.096	13.176^***^	0.776		

*** *p < 0.001; N = 344*.

*CR, Composite Reliability; AVE, Average Variance Extracted*.

*χ^2^ = 451.73; df = 387; χ^2^/df = 1.17; CFI = 0.99; GFI = 0.93; TLI = 0.99; RMSEA = 0.02*.

**Table 3:** Point estimate and Confidence Interval for Cronbach's alpha.

	**Point estimate**	**S.E**.	***T*-value**	**95% CI**	
				**Low**	**Up**
IA	0.901	0.091	9.953	0.724	1.079
UNC	0.828	0.163	5.071	0.508	1.148
SCA	0.844	0.116	7.283	0.617	1.071
FV	0.861	0.034	25.064	0.794	0.929
HV	0.850	0.248	3.422	0.363	1.336
EV	0.892	0.142	6.282	0.614	1.170
PB	0.819	0.239	3.426	0.351	1.288

**Table 4:** Means, standard deviations and correlations of variables.

	**Mean**	**SD**	**IA**	**UNC**	**SCA**	**FV**	**HV**	**EV**	**PB**
IA	2.606	1.040	**0.804**						
UNC	3.436	0.932	0.183	**0.729**					
SCA	4.048	0.668	0.064	−0.051	**0.750**				
FV	3.586	0.747	0.202	−0.331	0.362	**0.708**			
HV	3.391	1.012	0.243	−0.245	0.376	0.628	**0.810**		
EV	3.023	0.997	0.320	−0.179	0.292	0.521	0.638	**0.825**	
PB	3.270	0.897	0.055	−0.247	0.424	0.476	0.486	0.439	**0.776**

*The square root of AVE for discriminant validity is illustrated in bold font*.

In order to test the measurement invariance across males and females, according to the suggestions of Van De Schoot et al. (2015) and Deng et al. (2008), we conducted an invariance analysis of two subgroups, male and female. As shown in Table 5, in the factor loadings invariant model (model 6), the χ^2^ difference of 26.67 for 23 degrees of freedom was non-significant (*p* = 0.270), suggesting that item-factor loadings across males and females were equivalent. In addition, the χ^2^ difference of 31.81 for 30 degrees of freedom was non-significant (*p* = 0.376) in the model 7, the χ^2^ difference of 39.24 for 28 degrees of freedom was non-significant (*p* = 0.077) in the model 8 and the χ^2^ difference of 38.53 for 30 degrees of freedom was non-significant (*p* = 0.137) in the model 9, suggesting that measurement intercepts, covariances and measurement residuals across males and females were all equivalent.

**Table 5:** Invariance analysis across males and females in the measurement model.

**Model description**	**χ^**2**^**	**df**	**TLI**	**IFI**	**RMSEA**	**Δχ^**2**^**	**Δdf**	***p*-value**
Model 1: Combined baseline models (males and females)	1,587.629	768	0.863	0.865	0.056			
Model 2: Factor loadings constrained equal	1,614.296	791	0.862	0.864	0.055			
Model 3: Factor loadings and intercepts constrained equal	1,646.106	821	0.862	0.863	0.054			
Model 4: Factor loadings, intercepts and covariances constrained equal	1,685.342	849	0.860	0.860	0.054			
Model 5: Factor loadings, intercepts, covariances, and residuals constrained equal	1,723.871	879	0.858	0.858	0.053			
Model 6: Factor loadings invariant (Model 2 - Model 1)						26.67	23	0.270
Model 7: Measurement intercepts invariant (Model 3 – Model 2)						31.81	30	0.376
Model 8: Covariances invariant (Model 4 – Model 3)						39.24	28	0.077
Model 9: Measurement residuals invariant (Model 5 – Model 4)						38.53	30	0.137

*Δχ^2^ = difference in chi-square values; Δdf = difference in degrees of freedom*.

### Structural Model

**Figure 2:**
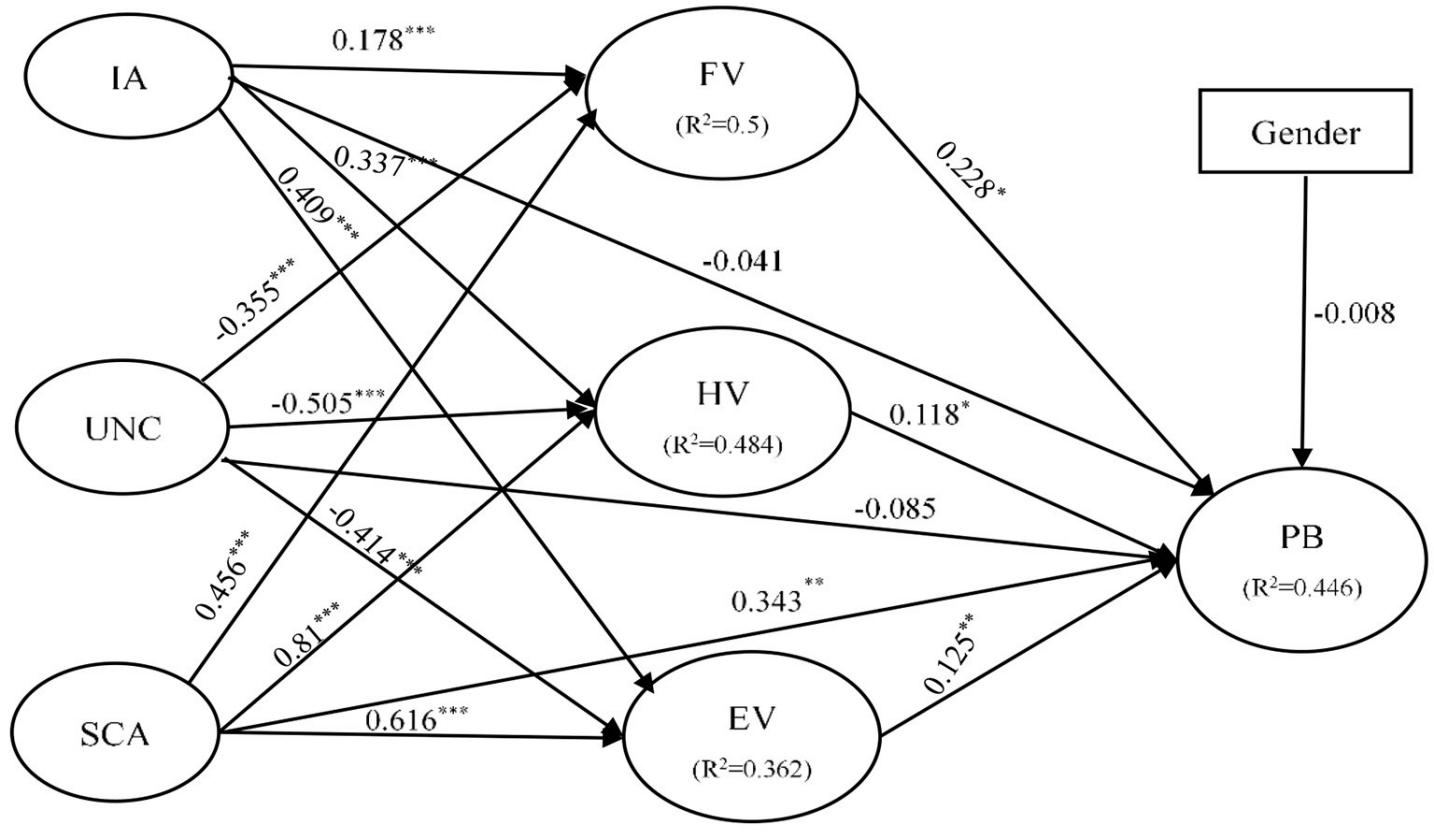
Tested model. (1) *p < 0.05; **p < 0.01; ***p < 0.001. (2) χ^2^ = 583.87; df = 509; χ^2^/df = 1.15; CFI = 0.99; GFI = 0.91; TLI = 0.99; RMSEA = 0.02.

This study used SEM to test the relationships between variables, including control variables (i.e., age, income, and education). According to the Bollen–Stine bootstrap to correct for bias in the model fit statistic, the resulting fit indices (χ^2^ = 583.87; df = 509; χ^2^/df = 1.15; CFI = 0.99; GFI = 0.91; TLI = 0.99; RMSEA = 0.02) were all acceptable. The structural model elucidated 44.6% of the variance of purchase behavior (see Figure 2).

As shown in Figure 2 and Table 6, the result of the hypothesis testing results indicates that 13 hypotheses were supported (H1a-H1c, H2a-H2c, H3a-H3d, H4, H5, and H6). Notably, IA (H1a: β = 0.178, *p* < 0.001; H1b: β = 0.337, *p* < 0.001; H1c: β = 0.409, *p* < 0.001) showed a significant influence on FV, HV, and EV respectively, supporting H1a, H1b, and H1c. And UNC (H2a: β = −0.355, *p* < 0.001; H2b: β = −0.505, *p* < 0.001; H2c: β = −0.414, *p* < 0.001) showed a significant impact on FV, HV and EV respectively, supporting H2a, H2b, and H2c. Meanwhile, SCA had a significant influence on FV, HV and EV respectively. Thus, H3a-H3c were supported. In addition, SCA, FV, HV, and EV had a significant impact on PB at a significant level of 1, 5, 5, 1% respectively, supporting H3d, H4, H5, and H6. However, IA, UNC and Gender had no significant effects on PB. Therefore, H1d, H2d, and H10 were not supported.

**Table 6:** Confirmation of the hypotheses.

**Hypothesis**	**Path**	**β**	**S.E**.	***t*-value**	***p*-value**	**Supported**
H1a	IA → FV	0.178	0.032	5.489	^***^	Yes
H1b	IA → HV	0.337	0.053	6.360	^***^	Yes
H1c	IA → EV	0.409	0.056	7.274	^***^	Yes
H1d	IA → PB	−0.041	0.053	−0.782	0.434	No
H2a	UNC → FV	−0.355	0.050	−7.102	^***^	Yes
H2b	UNC → HV	−0.505	0.073	−6.878	^***^	Yes
H2c	UNC → EV	−0.414	0.073	−5.669	^***^	Yes
H2d	UNC → PB	−0.085	0.078	−1.094	0.274	No
H3a	SCA → FV	0.456	0.068	6.700	^***^	Yes
H3b	SCA → HV	0.810	0.107	7.541	^***^	Yes
H3c	SCA → EV	0.616	0.104	5.923	^***^	Yes
H3d	SCA → PB	0.343	0.111	3.077	^**^	Yes
H4	FV → PB	0.228	0.102	2.234	^*^	Yes
H5	HV → PB	0.118	0.059	1.993	^*^	Yes
H6	EV → PB	0.125	0.047	2.643	^**^	Yes
H10	Gender → PB	−0.008	0.065	-0.128	0.898	No

* *p < 0.05*;

** *p < 0.01*;

*** *p < 0.001*.

In order to test the structural invariance across males and females, according to the suggestions of Van De Schoot et al. (2015), we conducted an invariance analysis of two subgroups, male and female. As shown in Table 7, the χ^2^ difference of 23.808 for 15 degrees of freedom was non-significant (*p* = 0.068) in the model 5 and the χ^2^ difference of 4.116 for 4 degrees of freedom was non-significant (*p* = 0.391) in the model 7, suggesting that path coefficients and structural residuals across males and females were all equivalent. However, the χ^2^ difference of 18.909 for 6 degrees of freedom was significant (*p* = 0.004) in the model 6, thus the structural covariances was not equivalent.

**Table 7:** Invariance analysis across males and females in the structural model.

**Model description**	**χ^**2**^**	**df**	**TLI**	**IFI**	**RMSEA**	**Δχ^**2**^**	**Δdf**	***p*-value**
Model 1: Combined baseline models (males and females)	1,746.941	774	0.817	0.840	0.061			
Model 2: Path coefficients constrained equal	1,770.749	789	0.819	0.838	0.06			
Model 3: Path coefficients and covariances constrained equal	1,789.658	795	0.818	0.835	0.06			
Model 4: Path coefficients, covariances and residuals constrained equal	1,793.774	799	0.818	0.835	0.06			
Model 5: Path coefficients invariant(Model 2 - Model 1)						23.808	15	0.068
Model 6: Structural covariances invariant (Model 3 – Model 2)						18.909	6	0.004
Model 7: Structural residuals invariant (Model 4 – Model 3)						4.116	4	0.391

*Δχ^2^ = difference in chi-square values; Δdf = difference in degrees of freedom*.

In order to further test the difference of consumers' gender on organic purchasing behavior, we conducted a group analysis of two subgroups, male and female. As shown in Table 8, SCA (β = 0.632, *p* < 0.01) showed a significant impact on PB in the male group. However, SCA had no significant effects on PB in female group. Meanwhile, EV (β = 0.144, *p* < 0.05) showed a significant impact on PB in female group. However, EV had no significant effects on PB in male group.

**Table 8:** Coefficient of group analysis.

**Hypothesis**	**Path**	**Gender**					
		**Male**			**Female**		
		**β**	**S.E**.	***p*-value**	**β**	**S.E**.	***p*-value**
H1a	IA → FV	0.149	0.039	^***^	0.214	0.055	^***^
H1b	IA → HV	0.237	0.060	^***^	0.490	0.096	^***^
H1c	IA → EV	0.343	0.067	^***^	0.538	0.097	^***^
H1d	IA → PB	−0.017	0.062	0.788	−0.104	0.099	0.292
H2a	UNC → FV	−0.312	0.071	^***^	−0.407	0.070	^***^
H2b	UNC → HV	−0.372	0.099	^***^	−0.650	0.106	^***^
H2c	UNC → EV	−0.224	0.101	^*^	−0.572	0.102	^***^
H2d	UNC → PB	−0.136	0.099	0.170	0.010	0.129	0.936
H3a	SCA → FV	0.536	0.133	^***^	0.437	0.078	^***^
H3b	SCA → HV	1.095	0.225	^***^	0.649	0.118	^***^
H3c	SCA → EV	1.151	0.237	^***^	0.343	0.111	^**^
H3d	SCA → PB	0.632	0.240	^**^	0.169	0.128	0.187
H4	FV → PB	0.196	0.139	0.158	0.270	0.159	0.090
H5	HV → PB	0.138	0.087	0.114	0.147	0.083	0.076
H6	EV → PB	0.061	0.069	0.377	0.144	0.070	^*^

* *p < 0.05*;

** *p < 0.01*;

*** *p < 0.001*.

### The Mediating Effect of Perceived Values

In order to investigate the indirect effects of the independent variable through the mediator, bias-corrected percentile bootstrapping and percentile bootstrapping were performed at a 95% confidence interval with 5,000 bootstrap samples (Taylor et al., 2007). Following the suggestions of Preacher and Hayes (2008), the confidence interval of the lower and upper bounds was calculated to test whether the indirect effects were significant. As shown in Table 9, the results of the bootstrap test showed that the total effect (total effect = 0.09, *p* < 0.05) and indirect effect (indirect effect = 0.051, *p* < 0.01) of IA on PB, and the total effect (total effect = −0.277, *p* < 0.001) and indirect effect (indirect = 0.077, *p* < 0.01) of UNC on PB were all significant, while the direct effects of those were no longer significant. The above findings indicated that EV played a completely mediating role in the relationships between IA and PB (H9a), and between UNC and PB (H9b). Thus, both H9a and H9b were supported. In addition, the total effect (total effect = 0.619, *p* < 0.001), indirect effect (indirect = 0.077, *p* < 0.01) and direct effect (direct effect = 0.343, *p* < 0.001) of SCA on PB were all significant. The above findings indicated that EV played a partially mediating role in the relationships between SCA and PB (H9c). Thus, H9c was supported. However, FV and HV had no mediating effects on the relationships between IA and PB, between UNC and PB, and between SCA and PB. Thus, H7a-H7c and H8a-H8c were not supported.

**Table 9:** Total effects, indirect effects, and direct effects of the model.

	**Point estimate**	**Product of coefficients**		**Booststrapping**					
				**Bias-corrected percentile 95% CI**			**Percentile 95% CI**		
		**S.E**.	***Z***	**Lower**	**Upper**	**Two-tailed significance**	**Lower**	**Upper**	**Two-tailed significance**
**Total effect**									
IA → PB	0.090	0.044	2.045	0.007	0.176	*	0.004	0.175	^*^
UNC → PB	−0.277	0.064	−4.328	−0.420	−0.167	^***^	−0.416	−0.163	^***^
SCA → PB	0.619	0.126	4.913	0.414	0.908	^***^	0.415	0.909	^***^
**Indirect effect**									
IA → FV → PB	0.041	0.023	1.783	0.005	0.103	^*^	−0.004	0.089	0.072
IA → HV → PB	0.040	0.027	1.481	−0.006	0.101	0.098	−0.006	0.101	0.097
IA → EV → PB	0.051	0.025	2.040	0.010	0.114	^**^	0.004	0.106	^**^
UNC → FV → PB	−0.081	0.045	−1.800	−0.183	−0.003	^*^	−0.172	0.008	0.072
UNC → HV → PB	−0.059	0.040	−1.475	−0.148	0.012	0.104	−0.150	0.010	0.097
UNC → EV → PB	0.077	0.038	2.026	0.015	0.169	^**^	0.007	0.155	^**^
SCA → FV → PB	0.104	0.058	1.793	0.006	0.243	^*^	−0.010	0.222	0.072
SCA → HV → PB	0.095	0.061	1.557	−0.021	0.219	0.110	−0.017	0.222	0.097
SCA → EV → PB	0.077	0.038	2.026	0.015	0.169	^**^	0.007	0.155	^**^
**Direct effect**									
IA → PB	−0.041	0.056	−0.732	−0.158	0.061	0.423	−0.156	0.064	0.450
UNC → PB	−0.085	0.075	−1.133	−0.234	0.062	0.257	−0.240	0.056	0.231
SCA → PB	0.343	0.125	2.744	0.129	0.623	^***^	0.136	0.639	^***^

*Estimation of 5,000 bootstrap sample*;

* *p < 0.05*;

** *p < 0.01*;

*** *p < 0.001*.

*Mediators: FV, HV, and EV*.

## Discussion

Using the SOR theoretical model and information similarity effect, we studied the relationships between information anxiety, uncertainty, sustainable consumption attitude, and organic purchasing behavior under the mediating role of their perceived values. And we used SEM to verify the research hypotheses. The main findings of the current study are as follows.

Firstly, regarding consumers' external similarity, the study results found that information anxiety and uncertainty had significant influences on perceived value (i.e., functional value, health value, and environmental value). Our findings are consistent with previous literature. Namely, Ruiz Mafé and Sanz Blas (2006) argued that information anxiety had a positive influence on consumers' perceived values and Nuttavuthisit and Thogersen (2017) argued that uncertainty toward the real attributes of organic food has a negative influence on consumers' perceived values. In addition, regarding consumers' internal similarity, the study results found that sustainable consumption attitude had significant and positive influences on perceived values and purchase behavior, which is in line with the results of prior literature (Follows and Jobber, 2000; Marchand and Walker, 2008; Lin and Huang, 2012). In other words, consumers with higher sustainable consumption attitude have higher perceived values of organic food. And they are more likely to buy organic food. However, information anxiety and uncertainty had no effects on organic purchasing, which is inconsistent with previous research results (Ruiz Mafé and Sanz Blas, 2006; Shiu et al., 2011). Two possible reasons are that, on the one hand, although consumers are eager to seek safe and high-quality food under the influence of COVID-19, many consumers do not trust organic food because the organic market in China is in its infancy (Xu, 2017). Moreover, Suki (2015) and Vega-Zamora et al. (2019) pointed out that trust is the basis of establishing exchange relationships between organic food buyers and sellers. This may explain why information anxiety did not have a significant effect on organic purchasing. On the other hand, although many Chinese consumers are uncertain about the information of organic food, the price of organic food is 2–4 times that of traditional food. Moreover, Xu (2017) believed that, to a large extent, the price of food represents the quality of food. Therefore, although some consumers are uncertain about the information of organic food, they may buy organic food according to the price of organic food. Thus, this may explain why uncertainty did not have a significant and negative effect on organic purchasing.

Secondly, regarding perceived values, the study results found that functional value, health value and environmental value had significant and positive influences on purchase behavior. Our findings are in line with the results of prior literature, which argued perceived values are considered important predictors of consumer decision-making (Sheth et al., 1991). It is worth noting that in the context of COVID-19, functional value, health value and environmental value all play an important role in organic purchase, which implies that consumers are more inclined to buy environmental and healthy organic food.

Thirdly, environmental value played mediating effects in the relationships between organic purchasing behavior and consumers' similarity (i.e., information anxiety, uncertainty, and sustainable consumption attitude). As we expected, there is a potential relationship among consumers' similarity, environmental value and purchase behavior. However, functional value and health value did not play mediating effects in the relationships between organic purchasing behavior and consumers' similarity (i.e., information anxiety, uncertainty and sustainable consumption attitude). This is not in line with our prediction. One possible reason is that some studies pointed out that COVID-19 is caused by ecological imbalance (Lvov and Alkhovsky, 2020), which prompts people to pay more attention to ecological balance and to pursue more environmentally friendly consumption. However, as the organic market in China is in its infancy, many consumers do not fully understand the benefits of organic food, especially its functional value and health value.

Finally, the results showed that gender had no significant relationship with purchase behavior. This is inconsistent with prior studies (Stobbelaar et al., 2007). Namely, Stobbelaar et al. (2007) believed that women prefer organic products more than men do. One possible reason is that due to environmental problems, the public is increasingly concerned about more environmentally friendly organic food (Basha and Lal, 2019). Therefore, consumers with different genders may all attach great importance to environmental protection. In addition, the results showed that there were gender differences in the impact of sustainable consumption attitude and environmental value on organic purchasing behavior. Our findings are in line with the results of prior literature, which argued the impact of consumer's gender differences on behavior and attitude is more and more obvious in the context of COVID-19 (Newburn, 2020; Stevens, 2020).

## Conclusion

This current study investigated the relationships between consumers' similarity (i.e., information anxiety, uncertainty, and sustainable consumption attitude), perceived values (i.e., functional value, health value, and environmental value) and organic purchasing behavior based on the SOR theoretical model and information similarity effect. And considering gender differences in consumers' similarity, perceived values, and organic purchasing behavior. Meanwhile, the mediating effects of perceived values on the relationship between consumers' similarity and purchasing behavior were also discussed, considering the background of COVID-19. The results indicated the significant association of information anxiety, uncertainty and sustainable consumption attitude with perceived values. And perceived values and sustainable consumption attitude had a positively significant influence on purchase behavior. In addition, environmental value played mediating effects in the relationships between organic purchasing behavior and information anxiety, uncertainty and sustainable consumption attitude. And the impact of sustainable consumption attitude and environmental value on organic purchasing behavior differed in gender.

The results provide valuable findings for the sustainable development of the organic food industry and may enrich the research in this field. The following insights can be obtained from the research results of this paper. First, Perceived values (i.e., functional value, health value, and environmental value) are important factors affecting consumers' organic purchasing behavior. Therefore, organic sellers and public policy makers can formulate corresponding strategies to drive consumers' organic consumption demand. That is to say, organic sellers should pay attention to specific consumer groups and improve consumers' perceived values of organic food products by maintaining customer satisfaction and pleasure. At the same time, the public policy makers should establish the concept of organic cultivation and breeding which is conducive to ecological civilization and human health through education and publicity, and enhance the correct values of consumers, so as to promote the development of organic industry.

Second, the results showed that consumers' uncertainty about organic food hinders consumers' perceived values of organic food, thus hindering organic consumption. Therefore, organic producers, organic sellers, and public policy makers should formulate corresponding strategies to promote consumers' understanding of organic information. Organic producers can show more production details to consumers through media and social platforms, and organic sellers can vigorously promote organic food through media and social platforms. At the same time, public policy makers can also provide more authoritative organic information to consumers through media and social platforms, so as to achieve the goal of promoting organic consumption and sustainable development.

Third, the results showed that information anxiety, uncertainty and sustainable consumption attitude have significant effects on organic purchasing behavior through environmental value. In particular, uncertainty positively affects organic buying behavior through environmental values. Therefore, in order to promote the consumption of organic food, the government needs to strengthen the publicity and education of environmental values, so as to promote the people to form values of protecting the environment.

## Limitations and Further Research

Some limitations of this study suggest directions for further research. First, the survey data were collected from organic consumers in China's first-tier cities, which may limit the generalizability of the study findings to other contexts. Future studies may be extended to other cities in China, which might help deepen the understanding of organic purchasing behavior and may further improve the generalizability of the study findings. Secondly, this paper only considered the similarity factors of information anxiety, uncertainty and sustainable consumption attitude, and may ignore other similarity factors (such as preference and personality characteristics), especially in the context of COVID-19. Future studies may consider more other similarity factors and may further enrich the results.

## Data Availability Statement

The original contributions presented in the study are included in the article/Supplementary Material, further inquiries can be directed to the corresponding author/s.

## Ethics Statement

Ethical review and approval was not required for the study on human participants in accordance with the local legislation and institutional requirements. Written informed consent for participation was not required for this study in accordance with the national legislation and the institutional requirements.

## Author Contributions

CL shaped the theoretical design. YZ was responsible for the statistical analysis. DC was responsible for the final composition. All authors contributed to the article and approved the submitted version.

## Funding

This paper was supported by the National Natural Science Foundation of China (NSFC, Grant Number: 71663038, 72064027).

## Conflict of Interest

The authors declare that the research was conducted in the absence of any commercial or financial relationships that could be construed as a potential conflict of interest.

## Publisher's Note

All claims expressed in this article are solely those of the authors and do not necessarily represent those of their affiliated organizations, or those of the publisher, the editors and the reviewers. Any product that may be evaluated in this article, or claim that may be made by its manufacturer, is not guaranteed or endorsed by the publisher.

## Appendix A

### Appendix A.1. Information Anxiety

IA1: After the COVID-19 outbreak, I often feel fear in the face of information about massive COVID-19.
IA2: After the COVID-19 outbreak, I get upset easily or feel panicky in the face of information about massive COVID-19.
IA3: After the COVID-19 outbreak, I sometimes feel like I'm falling apart and going to pieces in the face of information about massive COVID-19.
IA4: After the COVID-19 outbreak, I often feel anxious in the face of information about massive COVID-19.
IA5: After the COVID-19 outbreak, I can sometimes feel my heart beating fast in the face of information about massive COVID-19.

### Appendix A.2. Uncertainty

UNC1: I'm not sure about the authenticity of organic food labels.
UNC2: I'm worried that some of the claimed organic foods are not actually organic foods.
UNC3: I am worried that I may have spent more money on organic food.
UNC4: I don't have much confidence in the organic food on the market at present.

### Appendix A.3. Sustainable Consumption Attitude

SCA1: I think sustainable consumption is very important.
SCA2: I am very interested in sustainable consumption.
SCA3: I am very keen to search and read information about sustainable consumption.
SCA4: I am very concerned about sustainable consumption.

### Appendix A.4. Functional Value

FV1: Organic food has consistent quality.
FV2: Organic food is well made.
FV3: Organic food has an acceptable standard of quality.
FV4: Organic food would perform consistently.
FV5: Organic food is reasonably priced.
FV6: Organic food offers value for money.
FV7: Organic food is a good product for the price.

### Appendix A.5. Health Value

HV1: Organic food keeps my healthy.
HV2: Organic food is nutritious.
HV3: Organic food is high in protein.

### Appendix A.6. Environmental Value

EV1: The presence of resource shortage and environmental pollution has threatened life on earth.
EV2: The balance of nature is very delicate and easily upset.
EV3: We are approaching the limit the earth can sustain.
EV4: If things continue is the present course, the sustainability of the environment and future generations are highly threatened.

### Appendix A.7. Purchase Behavior

PB1: How often do you buy organic food in the organic food store or supermarket?
PB2: How often do you buy organic food online?
PB3: How often do you buy organic food on the community-group buying platform?
